# Restricting mutualistic partners to enforce trade reliance

**DOI:** 10.1038/ncomms10322

**Published:** 2016-01-27

**Authors:** Gregory A. K. Wyatt, E. Toby Kiers, Andy Gardner, Stuart A. West

**Affiliations:** 1 Department of Zoology, University of Oxford, South Parks Road, Oxford OX1 3PS, UK; 2 Institute of Ecological Sciences, Faculty of Earth and Life Sciences, Vrije Universiteit, De Boelelaan 1085, 1081 HV Amsterdam, The Netherlands; 3 School of Biology, University of St Andrews, Dyers Brae, St Andrews KY16 9TH, UK; 4 Magdalen College, Oxford OX1 4AU, UK

## Abstract

Mutualisms are cooperative interactions between members of different species, often involving the trade of resources. Here, we suggest that otherwise-cooperative mutualists might be able to gain a benefit from actively restricting their partners’ ability to obtain resources directly, hampering the ability of the restricted partner to survive and/or reproduce without the help of the restricting mutualist. We show that (i) restriction can be favoured when it makes the resources of the restricting individual more valuable to their partner, and thus allows them to receive more favourable terms of trade; (ii) restriction maintains cooperation in conditions where cooperative behaviour would otherwise collapse; and (iii) restriction can lead to either an increase or decrease in a restricted individual’s fitness. We discuss the applicability of this scenario to mutualisms such as those between plants and mycorrhizal fungi. These results identify a novel conflict in mutualisms as well as several public goods dilemmas, but also demonstrate how conflict can help maintain cooperation.

## Abstract (web summary)

Mutualistic interactions involve trading benefits between cooperative species. Here, the authors show that restricting the resource acquisition of a mutualistic partner can increase mutualism by promoting resource trade, which suggests that conflict may lead to increased cooperation.

## Graphical abstract

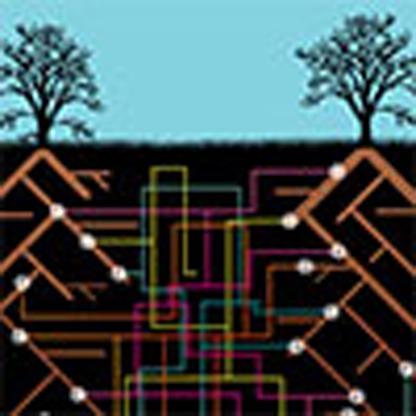

Mutualistic interactions are central to the survival and/or reproduction of most species. They provide essential ecosystem services such as pollination and seed dispersal, as well as constituting critical components of global carbon and nutrient cycles^1^,^2^. Mutualistic cooperation usually involves the different species trading either services or resources. For example: mycorrhizal fungi supply their host plants with phosphorous and other nutrients in exchange for host carbon; ants defend trees in exchange for food and housing; and flowering plants provide insects and birds with food in exchange for pollination services^3^,^4^,^5^.

Although these mutualisms are based on cooperative trade, there is an underlying tension because each partner is favoured to maximize its benefit from the interaction, leading to conflict and the potential for exploitation. In the extreme, non-cooperative cheats, who gain benefits from the cooperation of others without paying the cost of cooperation themselves, can even lead to the collapse of mutualisms^6^. At first glance, it seems unlikely that conflict could ever lead to increased cooperation in mutualisms. One possible way that this could happen is if mutualists could be favoured to restrict the resource acquisition pathways of their partners, in a way that made their partners more reliant on their mutualist to supply resources. In this case, conflict over the source of acquired resources could favour increased cooperation. However, the theoretical plausibility of this hypothesis remains unclear, as an actively restricting partner would incur costs, which could outweigh any benefits of increased trade.

We assess the theoretical feasibility of this hypothesis by modelling the interaction between two species. Our aim is to examine whether and why individuals would be favoured to restrict the resource acquisition ability of their mutualistic partners. Although we examine a relatively general model, we phrase it in terms of the interaction between plants and mycorrhizal fungi to provide biological grounding. We consider a costly trait that enables mycorrhizal fungi to restrict (decrease) the ability of their plant partners to directly take up phosphorus from the soil^7^. We first examine whether such a trait can be an evolutionarily stable strategy in mycorrhizal fungi. We then examine the consequences of restriction, for both the stability of the mutualism and the fitness of their restricted plant partners.

We found that mutualists can be selected to actively restrict their partners’ ability to directly obtain resources. This prediction arises because restriction increases the partner’s comparative advantage when engaging in the mutualism, and therefore improves the restricting individuals’ terms of trade. We further found that this restriction of a partner’s ability to directly obtain resources may maintain cooperation in conditions where cooperative behaviour would otherwise collapse, by enforcing reliance upon mutualistic partners. The combined effect of restriction and the maintenance of cooperation can lead to either an increase or a decrease in the restricted individual’s fitness. These results emphasize how mutualists will continue to be favoured to maximize their own fitness, even at a cost to their partner.

## Results

### Co-evolution of specialization and trade

We consider the co-evolution of strategies in both mycorrhizal fungi and plants. We assume that each plant has *n*_f_ fungal social partners, that each fungus has *n*_p_ plant social partners and that all individuals in a species are identical except for strategy. Both mycorrhizal fungi and plants require phosphorus and carbon for growth. We define an individual mycorrhizal fungus as a unit with high cooperation and low conflict, such that natural selection acts to maximize its inclusive fitness^8^,^9^. Most often, this will be the network that arises from a single fungal spore, as this network will likely be essentially clonal^10^,^11^. Each individual fungus has an initial supply of one unit of carbon and acquires a quantity *P*_f_ of phosphorus from the soil. The mycorrhizal fungus may restrict its plant partners, reducing the plants’ phosphorus uptake. A fungus investing an amount *r* into this restriction incurs a fitness cost, which reduces the fungus’s fitness to a fraction 1−*r* of what it would be in the absence of restriction. We assume that plants face a linear trade-off between the acquisition of carbon and phosphorus. That is, a plant that invests a proportion *x* of its acquisition effort into carbon acquisition obtains an amount *xC*_p_ of carbon and 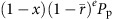 of phosphorus. *C*_p_ and *P*_p_ are the maximum availabilities of carbon and phosphorus, respectively, 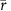 is the average restriction strategy of its fungal partners (phosphorus acquired diminishes as restriction increases), and the exponent *e*>0 modulates the cost effectiveness of the restricting trait (this form means that the cost effectiveness *e* has no effect when restriction is zero, as we would expect).

Fungi can only benefit from restriction through changes that the restriction causes in plant strategies. These changes must be immediate, otherwise restriction would always have a fitness cost to fungi when it first emerges^12^. Therefore, studying the evolution of restriction requires a model that takes into account phenotypic plasticity in plants, rather than just assuming a genetically fixed strategy (reviewed by McNamara^13^). A model that also incorporates the possibility for phenotypic plasticity in mycorrhizal fungi would add further biological realism but is technically challenging, because plants and fungi would respond to each other’s responses in a potentially infinite feedback loop. Therefore, we assume that fungal strategy is fixed over the course of the interaction, and hence its dynamics are over evolutionary timescales, but the plant’s allocation to trade and resource acquisition is dynamic over behavioural timescales, and it is the strategy underlying how it trades in response to partner cooperation that evolves.

Plants transfer a proportion *q*_p_ of their carbon to mycorrhizal fungi, while mycorrhizal fungi transfer a proportion *q*_f_ of their phosphorus to plants. We assume that plants and mycorrhizal fungi can discriminate among trading partners, as has been empirically demonstrated in this and other mutualisms^14^,^15^,^16^,^17^. We further assume that all individuals use a ‘linear proportional discrimination’ rule to divide resources among their partners, which means rewarding better trading partners by transferring more resources in proportion to the ratio in which they are received. For example, if a plant receives two-thirds of the benefits it acquires through trade from one mycorrhizal fungus and one-third from another, it sends two-thirds of the total carbon it allocates for trade to the former and one-third to the latter. Wyatt *et al.*^18^ have shown previously that linear proportional discrimination can be an evolutionarily stable strategy (ESS^19^,^20^) in trading mutualisms with the characteristics presented in this model.

We assume fitness functions *w*_p_=*C*_tp_^*a*^*P*_tp_^1−*a*^ for plants and *w*_f_=*C*_tf_^*b*^*P*_tf_^1−*b*^(1−*r*) for mycorrhizal fungi, where *C*_tp_, *P*_tp_, *C*_tf_ and *P*_tf_ are the total amounts of carbon and phosphorus acquired (after trade), and where 0<*a*<1 and 0<*b*<1 mediate the effects of carbon and phosphorus on fitness. These functions simplify our analysis by supposing that the effects of kin selection and local competition cancel^21^. In the Methods, we give explicit expressions for the total amounts of carbon and phosphorus acquired in terms of evolved strategies (*x*, *q*_p_, *q*_f_ and *r*) and model parameters (*C*_p_, *P*_p_, *P*_f_, *n*_p_, *n*_f_, *a*, *b* and *e*). We then calculate co-evolutionary ESSs for resource acquisition, allocation to trade and restriction (Methods, expressions (7a)–(7d), (8), (10), (13) and (15), 68, 75, 83). We do not list the expressions for the ESSs here, because the expressions are cumbersome and change in different ecological scenarios. Instead, we analyse some key properties of these ESSs below. We find that at the competitive equilibrium, all individuals in a species are predicted to adopt the same strategy.

### When it pays to restrict a partner’s access to resources

In the Methods (expressions (7a)–(7d), 43, 46, 47), we show that restriction occurs at the ESS (that is, >0 in the ESS expression) when two conditions are satisfied: (i) phosphorus restriction by the fungus is sufficiently cost effective; (ii) plants would otherwise obtain an appreciable amount of phosphorus directly from the soil. If conditions (i) and (ii) are both satisfied, then mycorrhizal fungi are favoured to restrict plants’ access to phosphorus. By doing this, they make phosphorus more valuable to plants, and are then able to get more carbon in exchange for the phosphorus that they trade.

Restriction is cost effective when *e*>*n*_f_/*b* (condition (i), above). Increasing the cost effectiveness of restriction (*e*) makes this condition less stringent, and hence promotes restriction. Increasing the number of mycorrhizal fungi per plant (*n*_f_) makes restriction less likely because a single fungus colonizes fewer of a plant’s roots, and therefore gets a lower individual payoff for unilateral restriction of plant phosphorus uptake. Decreasing the importance of carbon for fungal fitness (*b*) devalues trade for fungi, and therefore diminishes the likelihood that they invest in restricting to acquire more carbon per unit of phosphorus they trade.

Restriction is only favoured if plants would otherwise obtain an appreciable amount of phosphorus directly from the soil (condition (ii), above). If plant phosphorus acquisition were minimal relative to fungal phosphorus acquisition (*n*_p_*xP*_p_<<*n*_f_*P*_f_), then restricting this phosphorus uptake would have little influence on trading dynamics. Appreciable plant phosphorus acquisition requires sufficient carbon availability for plants. That is, plants must have enough carbon that selection favours re-allocating some resources away from acquiring carbon, and investing them into acquiring phosphorus directly. This occurs when




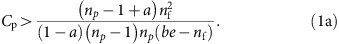




In addition, plants must be efficient at obtaining phosphorus directly from the soil, which is when




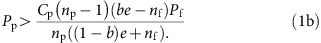




Our results can be understood through the lens of comparative advantage^22^. Comparative advantage is an economic framework for analysing trade. It predicts that both trader types can be better off if they specialize in acquiring a resource that they have a relative advantage in acquiring, and use that resource to trade for another. Individuals gain more from trade if the resource they acquire is scarcer, so they can benefit from restricting the access of other market participants to that resource^23^,^24^,^25^,^26^. However, in complex ecological systems, this may mean that resources flow out of the mutualistic system as a whole and go to non-mutualistic competitors. The potential cost of this depends on the degree of ecological versus intraspecific competition.

The restriction of a partner is a public good, as other mycorrhizal fungi interacting with a restricted plan gain from the plant’s increased reliance on trade for phosphorus. These benefits to other unrelated fungi are not taken into account by natural selection on a restricting fungus, so the level of restriction is always less than would be socially optimal from the perspective of fungi as a species. This can even lead to a failure to restrict even when all fungi would be better off if they all practised restriction, a public goods dilemma. This dilemma is exacerbated when plants have more fungal partners, as provision of the public good depends on a larger number of participants. This increases the incentive to free-ride. The public good nature of restriction also means that it could be more likely to be favoured if the different mycorrhizal fungi interacting with a single plant tended to be related^10^. However, local competition between fungi could mean that restriction is less likely to be favoured^10^,^21^,^27^.

### How restriction affects the stability of mutualistic trade

Theory suggests that hosts are under strong selection pressure to avoid cooperating with less-beneficial symbionts. Therefore, we might expect a breakdown in cooperation when fungi restrict the amount of phosphorus that plants can take up from the soil. In the Methods, we show that our model leads to the opposite result: allowing mycorrhizal fungi to restrict the direct phosphorus uptake pathway in plants maintaining cooperation under conditions where it otherwise would not be (expressions (7a)–(7d), 43, 46, 47).

When plants take up more phosphorus from the soil, they do not need to trade with mycorrhizal fungi to acquire phosphorus. This would lead to plants decreasing the carbon supply to their fungal partners. Because fungi have less carbon available to trade for, they are favoured to transfer less phosphorus. In turn, plants become even less reliant on fungal phosphorus, which favours them to decrease carbon transfers further. Without restriction, trade collapses altogether if the amount of phosphorus that plants can take up from the soil goes beyond the threshold




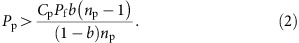




**Figure 1:**
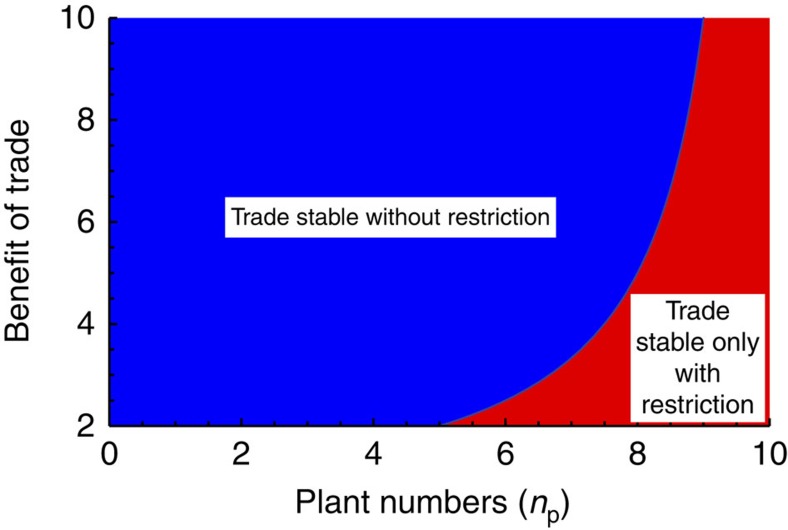
The effect of phosphorus restriction on the stability of mutualistic trade. In the blue region, mutualistic trade is favoured with or without fungal phosphorus restriction. In the red region, mutualistic trade is only favoured when we allow fungal phosphorus restriction. Restriction favours trade when it would otherwise collapse because the benefit of trade (relative increase in mutualism efficiency if plants rely more on trade for phosphorus) is too low.

However, we find that restriction can maintain trade in situations when it would otherwise collapse (Fig. 1). Restriction reduces the quantity of phosphorus that plants can take up from the soil, so they are favoured to increase trade with mycorrhizal fungi. The increased stability of mutualisms when each partner has greater control over one resource has already been recognized^18^,^28^—our result provides a novel mechanism by which this control can be achieved.

We might expect that decreased carbon availability would lead to increasing outbidding competiton by mycorrhizal fungi in trade. Instead, our model predicts that they allocate less phosphorus to trade. This is because selection optimises the level of fungal investment into mutualistic cooperation before the decrease in carbon availability. When carbon decreases, the returns for outbidding competition are lower, so they invest less phosphorus in trade.

### How restriction affects plant fitness

**Figure 2:**
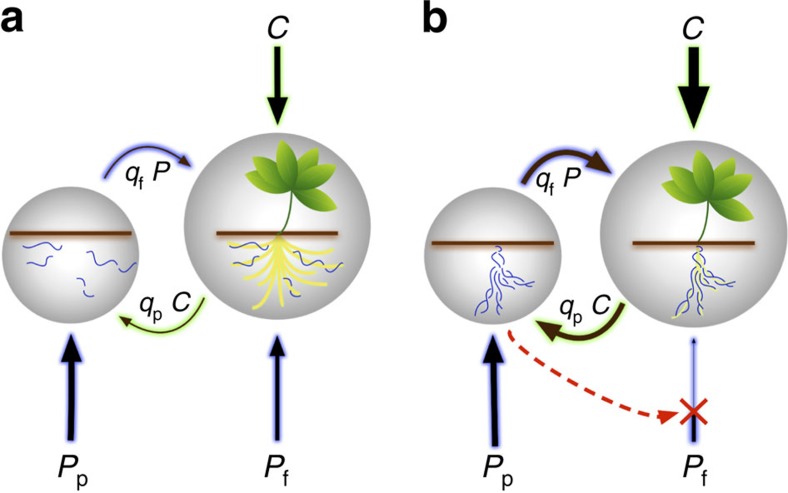
The effect of phosphorus restriction on the mutualistic system. (**a**) Plants acquire phosphorus and carbon, whereas mycorrhizal fungi acquire phosphorus. Plants trade carbon to mycorrhizal fungi in exchange for phosphorus. (**b**) Mycorrhizal fungi restrict the ability of plants to directly acquire phosphorus (red cross). This restriction makes plants reliant on fungal phosphorus, so plants invest more into acquiring carbon directly and trade more carbon to compete for phosporous. The increased flows of carbon in trade favour mycorrhizal fungi that transfer more phosphorus.

Restriction could potentially have positive or negative consequences for plant fitness. Restriction reduces phosphorus availability, so the first-order effect of restricting the resource acquisition pathway is to decrease plant fitness. However, phosphorus restriction also changes the terms of trade, which increases plant allocation to carbon acquisition (*x*; Fig. 2). This specialization can increase the efficiency of the mutualistic system as plants are relatively better at carbon acquisition, and so can provide a fitness benefit to the plants^22^.

**Figure 3:**
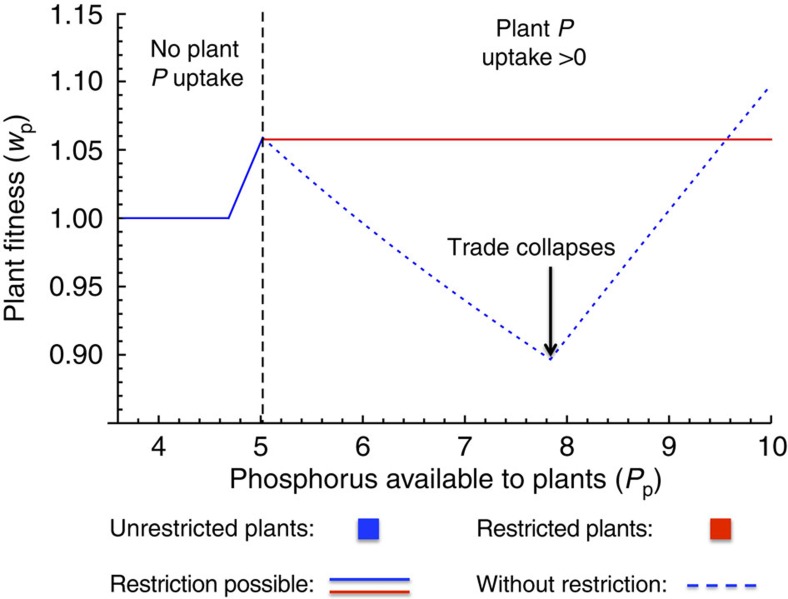
Plant fitness with and without restriction. The impact of fungal restrictions on plant fitness changes with the phosphorus available for direct uptake by plants (*P*_p_). When plants do not acquire any phosphorus, restrictions are not favoured and so have no impact (left of black dashed line). When restriction does not happen, phosphorus acquisition by competitor plants reduces carbon supply (downward sloping dotted line), and can mean that restricted plants would have higher fitness (as shown in graph). When restrictions are not possible, trade collapses and plant fitness recovers to exceed fitness without restriction as *P*_p_ increases (upward sloping dotted line). (In graph, *n*_p_=2, *n*_f_=12, *C*_p_=1.9, *P*_f_=5.5, *a*=0.17, *b*=0.6, *e*=110).

Overall, we find that the influence of restriction on plant fitness depends on the amount of phosphorus available for direct uptake by plants (Fig. 3). Although restriction usually decreases plant fitness, we find that in certain situations restriction can increase plant fitness. At low levels of phosphorus availability, restriction never evolves.

At intermediate phosphorus availability, when there would be trade even without restriction, the restriction increases plant fitness when




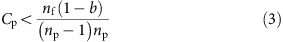




This requires two biological conditions to be satisfied. First, mycorrhizal fungi must be relatively less dependent on carbon obtained via its own trading interactions (small *C*_p_), such that only a small amount of trade would be favoured without restriction. Second, there must be few plants relative to fungal individuals (small *n*_p_, large *n*_f_). This means that an individual plant controls a large proportion of the tradable carbon. It also means that more fungi benefit from a single fungus’ restriction, thus increasing the public goods dilemma. This ensures that when sanctions are nonetheless favoured, they are at a much lower level than would be optimal from the perspective of mycorrhizal fungi as a whole. This means that they do not have an excessively negative impact on plant fitness. Without restriction, this individual plant places less value on phosphorus received in trade because it takes up more from the soil. Hence, its costs (decrease in total quantity of phosphorus received) when it decreases carbon supply in order to benefit by increasing the quantity of phosphorus it receives per unit of carbon (increasing the price of carbon in units of phosphorus paid) are lower. Each individual plant is favoured to adopt this strategy. However, it causes a public goods dilemma in plants. Their collective decrease in carbon traded favours less mutualistic cooperation in mycorrhizal fungi. That is, the fungi are favoured to transfer less phosphorus to plants. Restriction resolves this problem because it increases the individual costs to plants of decreasing carbon supply because they value fungal phosphorus more. The higher dependence on trade of restricted plants greatly increases total volumes traded, which benefits all partners.

When sufficient phosphorus is available for trade to collapse in the absence of restriction (inequality (2) is satisfied), plant fitness increases with restriction when




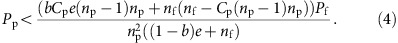




This increase in plant fitness despite resource losses owing to restriction occurs because restriction increases trade, as described above. However, at high levels of phosphorus availability to plants, it is increasingly difficult for fungal trade to compensate for the costs of restriction. At extremely high levels of phosphorus, trade with mycorrhizal fungi cannot compensate for restriction so the trait decreases plant fitness.

Even though plants may sometimes benefit from restriction, plants are never favoured to reduce their own direct phosphorus uptake. The cost to an individual plant of taking up less phosphorus from the soil would not be outweighed by increased phosphorus received in trade. The reason for this is that the phosphorus available in trade depends on the direct uptake of all plants that share a fungal partner, not just that of a single plant. Our model has no means for them to resolve this public goods problem. Another factor could be that mycorrhizal fungi can more efficiently control the supply of soil resources, but building in such complexity would require more empirical evidence on mechanistic detail.

Our model, where mutualistic cooperation is driven by market dynamics, makes an opposite prediction to models where mutualistic cooperation is driven by alignment of fitness interests^12^,^29^,^30^. We found that plant fitness increases with restriction only when each fungus interacts with a small number of plants. As discussed above, this is because less mutualistic cooperation is favoured in plants when there are fewer plant competitors, which decreases benefits returned. In contrast, other models predict that when there are fewer competitors in a species (higher relatedness), mutualistic cooperation increases^12^,^29^,^30^. In these models, benefits from the mutualism are shared, rather than competed for, and so cooperation is more favoured when benefits are shared between fewer individuals (or relatives).

**Figure 4:**
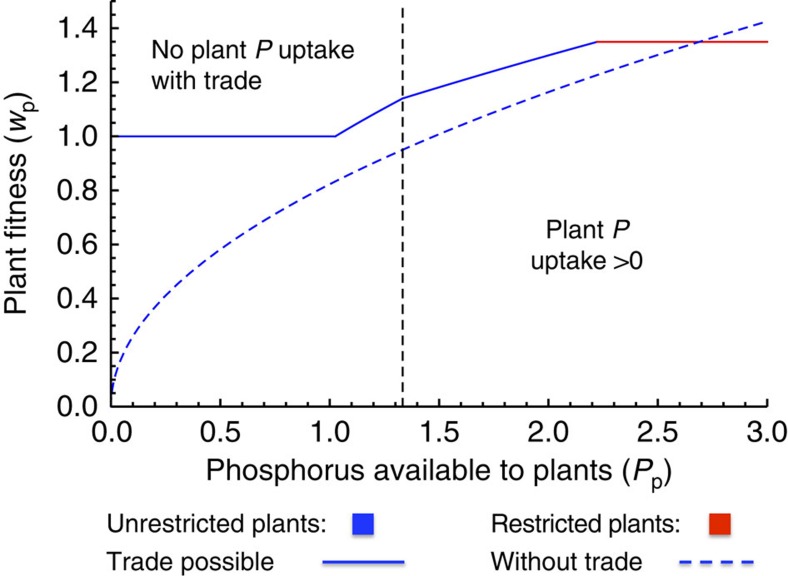
Plant fitness with and without trade. The impact of trade on plant fitness changes with phosphorus available for direct uptake by plants (*P*_p_). When plants do not directly acquire any phosphorus, trade increases plant fitness (left of black dashed line). When plants acquire phosphorus but mycorrhizal fungi are not favoured to restrict, trade also increase plant fitness (solid blue line right of dashed black line). When fungi begin to restrict, the restriction cancels out any beneficial effects of increases in *P*_p_ for plants (red line). Here, plants may be better off if they do not have trading partners. (In graph, *n*_p_=3, *n*_f_=3, *C*_p_=5, *P*_f_=2, *a*=0.5, *b*=0.5, *e*=12).

Do we expect plants to respond to restriction? They cannot be favoured to preferentially trade with non-restricting fungi, because trade is separate from the restricting trait. If they preferentially trade with non-restricting fungi, they are making suboptimal trades and this is not favoured by natural selection. Is it ever beneficial for plants to cut connections with their fungal partners completely? Our model suggests that when phosphorus available for direct uptake by plants is present in very high concentrations, plants without any fungal symbionts gain more phosphorus compared with plants that are colonized, but restricted, by fungal partners (Fig. 4). In these circumstances, interacting with mycorrhizal fungi leads to a fitness cost not benefit, and so if plants can completely terminate their interaction with their fungal partners, then they would be favoured to do so. A reduction in fungal colonization is sometimes observed under very high nutrient conditions^31^,^32^. However, in some cases, plants might not be able to completely cut their interactions with their fungal partners, which could lead to a fitness cost of interacting with fungi, termed mycorrhizal depression (Fig. 4)^7^,^28^,^33^.

## Discussion

How relevant is this model to mutualisms in nature? Mycorrhizal fungi appear to inhibit plants’ direct phosphorous uptake pathway via molecular suppression mechanisms^33^,^34^,^35^,^36^. Further evidence for restriction in mutualisms comes from the ant–acacia mutualism. Acacia trees have been shown to provide a protein food source that alters their mutualistic ants’ digestion, limiting the extent to which these ant partners can use other food sources^37^. Our model suggests that restriction mechanisms should be looked for in any trading mutualism where there is scope for a partner to increase their comparative advantage. More generally, we might also expect restriction within species when different classes of individual exchange benefits; for example, when a male harms a female to decrease her life expectancy and thus increase her immediate investment into reproduction with the harming male^38^,^39^,^40^.

We have shown that individuals can be favoured to restrict the quantity of resources directly acquired by their mutualistic partners to make their own supply of those resources more valuable. Human history has repeatedly proven how devastatingly effective such strategies can be, as demonstrated by the eradication of spice-producing trees in the East Indies by the Dutch colonial authorities. This eradication strategy restricted the ability of other European nations to acquire cloves, nutmeg and mace except through direct trade with the Dutch East India Company. Another example of restriction increasing comparative advantage is England’s embargo on textiles manufactured abroad^41^,^42^. This helped launch England’s textile industry by granting domestic producers exclusive access to the domestic market. These examples also help illustrate that restriction is most effective where restriction is under the control of a single agent (inequalities (3) and (4)). The efforts of taxi companies to lobby, strike and serve lawsuits against the introduction of both licensed and non-licensed (that is, rideshare) competitors are a more recent example of restriction. Here, the multitude of drivers and taxi licence owners that must act to make restriction effective does not prevent it occurring. However, in mutualisms where restricting individuals compete with their peers for trade, restriction may lead to increased reliance on trade by both partner species, and consequently promote cooperation. This can even stabilize mutualisms in circumstances under which they would otherwise break down.

## Methods

### Conditions for the evolution of trade and restriction

We derive expressions for the total amounts of carbon and phosphorus acquired (*C*_tp_, *P*_tp_, *C*_tf_ and *P*_tf_) in terms of evolved variables and model parameters. To do so, we first consider a focal plant with a strategy pair (*x*, *q*_p_), which may or may not be an ESS. This plant is in a nearly uniform population in each species with plant proportions of investment into carbon acquisition and allocations to trade clustered around 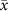 and 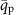, respectively, and where the fungal allocations to trade and investments in restriction are nearly uniform and clustered around 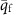 and 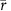, respectively. The focal plant acquires an amount *xC*_p_ of carbon and retains a fraction 1−*q*_p_ of this, so that it has a total quantity of carbon *C*_tp_=*xC*_p_(1−*q*_p_).

The plant also acquires an amount of phosphorus 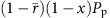 directly and receives an amount of phosphorus 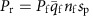 via trade, where 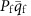 is the average quantity of phosphorus each mycorrhizal fungus trades, *n*_f_ is the total number of mycorrhizal fungi hosted by the plant, and the dummy variable *s*_p_ is the share of the traded phosphorus the focal plant acquires. We now consider how to substitute evolved variables and model parameters for the dummy variable *s*_p_. By linear proportional discrimination, plants acquire traded phosphorus based on the relative amount each provides. Thus, the focal plant’s share (*s*_p_) is the proportion of total carbon transferred that the focal plant provides. This is the total quantity that the plant transfers (*xC*_p_*q*_p_), divided by the total quantity provided by all competitors including the focal plant 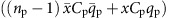. Hence, the proportion of total traded phosphorus that the plant acquires is 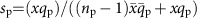. The total quantity of phosphorus available to plants is the sum of the quantity they take up from the soil and the quantity they acquire via trade, so 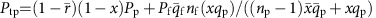.

Substituting the expressions for *C*_tp_ and *P*_tp_ that we have derived above into the plant’s fitness function given in the main text yields




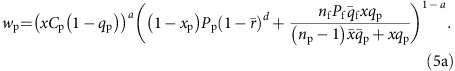




Similarly, the focal mycorrhizal fungus has an initial amount of carbon 1 and receives an amount of carbon 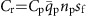 via trade, where 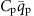 is the average quantity of phosphorus each mycorrhizal fungus trades, *n*_p_ is the total number of mycorrhizal fungi hosted by the plant and the dummy variable *s*_f_ is the share of the traded carbon that the focal fungus acquires. We now consider how to substitute evolved variables and model parameters for the dummy variable *s*_f_. By linear proportional discrimination, fungi acquire traded carbon based on the relative amount each provides. Thus, the focal fungus’ share (*s*_f_) is the proportion of total phosphorus transferred that the focal fungus provides. This is the total quantity that the fungus transfers (*P*_f_*q*_f_), divided by the total quantity provided by all competitors including the focal fungus 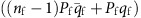. Hence, the proportion of total traded phosphorus that the plant acquires is 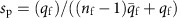. The total quantity of phosphorus available to plants is the sum of the quantity they take up from the soil and the quantity they acquire via trade, so 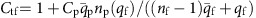.

The focal fungus also acquires an amount *P*_f_ of phosphorus and retains a fraction 1−*q*_f_ of this, so that it has a total quantity of phosphorus *P*_tf_=*P*_f_(1−*q*_f_). Substituting the expressions for *C*_tf_ and *P*_tf_, we have derived above into the fungus’ fitness function given in the main text yields




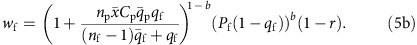




We use equations (5a) and (5b) to calculate evolutionary maxima. Specifically, we calculate ESSs, strategies where deviations from population mean strategies leads to decreased fitness (mathematically, we calculate: 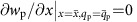 and 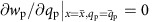 for plants, 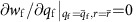 and 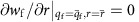 for mycorrhizal fungi). We find that the fitness equilibrium is always unique given parameter values and that the second derivatives of fitness with respect to individual strategy are always negative (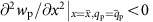 and 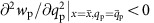 for plants, 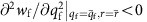 and 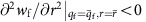 for mycorrhizal fungi). Therefore, the fitness equilibrium is a unique ESS. In this analysis, we do not consider whether the ESS is also convergence stable^43^,^44^,^45^,^46^. The ESSs for plants depend on the strategies used by fungi, and the ESSs for fungi depend on the strategies used by plants^20^. Hence, we need to find the ESSs for plants given fungal strategy (
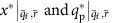
), and the ESSs for fungi given plant strategy (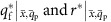). Then, we can solve these four resulting expressions to find co-evolved ESSs in terms of model parameters only (x*, *q*_p_*, *q*_f_*, r*). We first calculate the equilibria of fitness for allocation to direct carbon uptake and trade in a focal plant, *∂w*_p_/*∂x*_p_=*∂w*_p_/*∂q*_p_=0. At an ESS, any individual receives population mean fitness to first order, so we take the focal plant’s strategy to be the population average (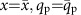). Substituting, we find that at equilibrium




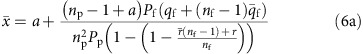




and




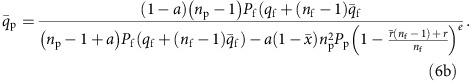




We use the expressions for 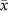 and 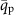 in equations (6a) and (6b) in the equation for fungal fitness equation (5b) and calculate the maxima with respect to allocation to trade and phosphorus restriction, *∂w*_f_/*∂q*_f_=*∂w*_f_/*∂r*=0. Again, at an ESS, any individual receives population mean fitness to first order, so we take the focal fungus’ strategy to be the population average (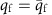, 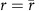). Substituting, we find that at a fixed point




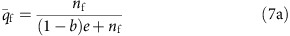




and




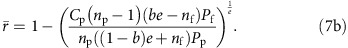




Using the expressions for 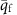 and 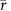 from equations (7a) and (7b) in equations (6a) and (6b), we find plant strategy as a function of model parameters only




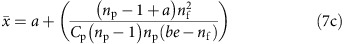







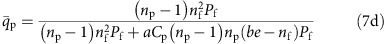




Therefore, equations (7a)–(7d), 43, 46, 47 specify a co-evolutionary ESS when each of 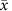, 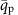, 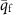 and 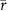 are between 0 and 1, as all values outside this range are not feasible strategies. The values of 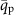 and 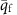 specified by equations (7a) and (7d) are always between 0 and 1. The value of 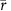 specified by equation (7b) is between 0 and 1 if *e*>*n*_f_/*b* and *P*_p_>*C*_p_(*n*_p_−1)(*be*−*n*_f_)*P*_f_/*n*_p_((1−*b*)*e*+*n*_f_), while the value of 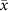 specified by equation (7c) is between 0 and 1 if 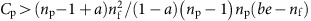. Hence, when these three inequalities are satisfied, equations (7a)–(7d), 43, 46, 47 are a co-evolutionary ESS.

We look for other strategies that are constrained maxima. That is, strategies where any alternative with higher fitness is not feasible (allocations <0 or >1). We eliminate 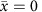 and 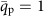 as this would leave plants without carbon, and 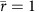 and 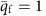 as this would leave mycorrhizal fungi with either zero fitness or no phosphorus. We first set 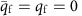 and work out the evolved best response values for the other variables if all other individuals use the same strategy:




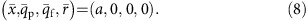




We verify whether fitness at *q*_f_=0 is greater than at *q*_f_>0, given the corresponding values of 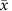, 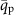 and 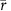:




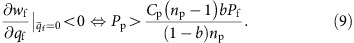




Hence, the strategies defined by expression (8) are a co-evolutionary ESS if the inequalities in expression (9) are satisfied. We then set 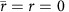 and find the condition for a co-evolutionary ESS by the same method with:




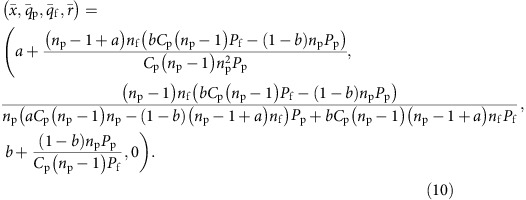




We verify whether fitness at *r*=0 is greater than at *r*>0, given the corresponding values of 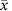, 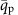 and 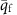:




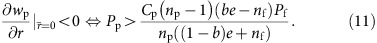




In the parameter region from expression (11), the best response values in equation (10) are feasible (and equation (10) is thus a co-evolutionary ESS) if




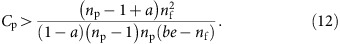




Finally, we set 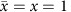. In this case, the phenotype of plants may or may not respond to a small change in fungal strategy, depending on parameter values. We first suppose that it does not, so




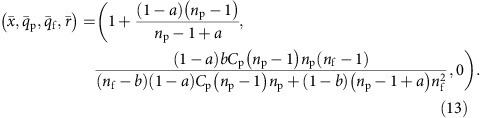




Now, we verify whether fitness at *x*<1 is indeed greater than at *x*=1, given the corresponding values of 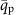, 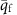 and 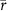:




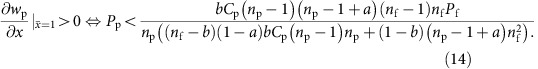




Hence, plant phenotype does not deviate from 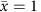 when fungal strategy is at its optimum and equation (13) is thus a co-evolutionary ESS whenever parameter values satisfy inequality (14).

If instead the phenotype of plants responds to fungal strategy when 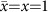, fungal strategy is set so that 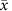 in equation (7d) is exactly 1:




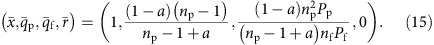




We verify whether fitness at 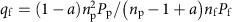 is a maximum, given the corresponding values of 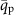, 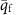 and 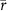:




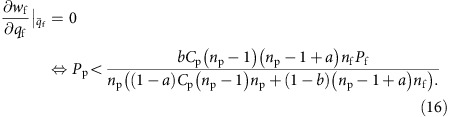




So, equation (15) is a co-evolutionary ESS whenever parameter values satisfy inequality (16), but not inequality (14). If inequality (14) is satisfied, plant phenotype would not deviate from 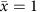 with a change in fungal strategy.

## Additional information

**How to cite this article**: Wyatt, G. A. K. *et al.* Restricting mutualistic partners to enforce trade reliance. *Nat. Commun.* 7:10322 doi: 10.1038/ncomms10322 (2016).
